# Remarkable Thermal Performance Enhancement of Micro Heat Pipes with Graphene-Nanoplatelet Nano-Wicks

**DOI:** 10.3390/nano13020232

**Published:** 2023-01-04

**Authors:** Jie Sheng Gan, Yew Mun Hung

**Affiliations:** Mechanical Engineering Discipline, School of Engineering, Monash University, Bandar Sunway 47500, Malaysia

**Keywords:** graphene nanoplatelets, film-wise evaporation, micro heat pipe, ultrafast water permeation

## Abstract

The ultrafast water permeation property of graphene nanoplatelets (GNPs) synergically enhances the evaporation and water circulation processes in a micro heat pipe (MHP). An MHP is a promising phase-change heat-transfer device capable of transferring large amounts of heat energy efficiently. The hydrophobic, atomically smooth carbon walls of GNPs nanostructures provide a network of nanocapillaries that allows water molecules to intercalate frictionlessly among the graphene layers. Together with the attraction force of the oxygenated functional groups, a series of hydrophobic and hydrophilic surfaces are formed that significantly improve the water circulation rate. The intercalation of water molecules encourages the formation of water-thin film for film-wise evaporation. The effect of nano-wick thickness on the thermal performance of the MHP is investigated. A thinner GNP nano-wick is more favorable to film-wise evaporation while a thicker nano-wick promotes a higher water circulation rate from the condenser to the evaporator, leading to the existence of an optimal thickness. By benchmarking with the uncoated MHP, the thermal conductance of an MHP with a 46.9-µm GNP nano-wick manifests a maximum enhancement of 128%. This study provides insights on the feasible implementation of GNP nano-wicks into a highly efficient micro-scale electronics cooling device for environmental sustainability.

## 1. Introduction

The single-layer honey-lattice arrangement of graphene and its derivatives allows remarkable physical and thermal properties [1,2,3,4]. Due to its high thermal conductivity, graphene and its derivatives manifest as excellent heat-conducting materials for electronics cooling applications [5,6,7,8,9,10]. However, there is a limitation to the thermal conductivity of graphene derivatives in these practical applications. The interaction between graphene layers is governed by weak Van der Waals bonds and the thermal conductivity is found to decrease as the graphene layer thickens. In the case of multilayer graphene, the thermal conductivity is found to drop as low as 10 W/m·K [5,11]. This significant drop in thermal conductivity has been the major hurdle in implementing graphene derivatives in thermal applications.

Over the years, phase-change heat transfer has gained significant recognition due to its high effectiveness in thermal management systems [12,13,14,15]. Instead of relying on the low heat-transfer coefficient of single-phase heat transfer such as conduction and convection [16,17], phase-change heat transfer utilizes the change of states (usually from liquid to vapour and vice versa) through evaporation and condensation processes. The phase-change heat transfer can be enhanced through surface modification [18,19,20,21]. According to the well-known Wenzel and Cassie-Baxter model, the surface wettability can be improved by altering the surface morphology [22]. Micro and nano-porous materials are commonly used to improve the surface wettability through methods such as sintering metal particles and nanoparticle deposition. Carbon nanomaterials such as graphene oxide (GO) [23,24,25,26], graphene nanoplatelets (GNPs) [19,20,21,27,28,29] and carbon nanotubes (CNTs) [30,31,32,33,34] are commonly used to improve the surface wettability. While the underlying principles are yet to be determined, the improved efficiency is generally attributed to the increase in heat-transfer surface area, improved surface wettability and capillary wicking due to the nanostructures [35].

The ultrafast water permeation property of graphene-based materials has been extensively utilized in various applications, such as desalination [36,37,38,39], nanofiltration [40,41,42,43] and phase-change heat transfer [9,15,29] systems. Among the carbon nanomaterials, the GNPs are found to exhibit the most superior ultrafast water permeation property [44,45,46]. GNPs consist of multilayers of exfoliated graphene nanosheets with the oxygenated functional groups—such as hydroxyl, epoxy, carbonyl, carboxyl groups—attached at their periphery [6,44,45,46]. The hydrophilic oxygenated functional groups act as the driving-force source of the ultrafast water permeation property, which generates the intercalation of water molecules through the carbon nanostructures. In the non-oxidized regions, vacant spaces formed between graphene nanosheets and the ultrafast water permeation is attributed to the near-frictionless interaction between the hydrophobic carbon wall and the well-ordered hydrogen bonds of water molecules [46,47]. Therefore, the atomic-scale capillaries and cavities allow ultrafast water transport induced by the high capillary pressures, and large slip lengths are formed in the GNP nanostructures. Together with the tortuous nano-porous structures of GNPs, the intercalation of water molecules through the GNP interlayers leads to the formation of an ultrathin water film and encourages film-wise evaporation, which is more effective than bulk evaporation [21,48]. The latent heat of vaporization is absorbed through the heated surface and film-wise evaporation occurs throughout the GNP surface. While graphene nanostructures increase the heat-transfer surface area significantly, a larger nucleation site density, increased bubble frequency and smaller bubble diameter can be obtained. This ultrafast water transport property of graphene serves a dual purpose: enhancing the circulation rate of water via a nanocapillary effect and inducing the film-wise evaporation due to the formation of ultrathin water film [7,29].

**Figure 1:**
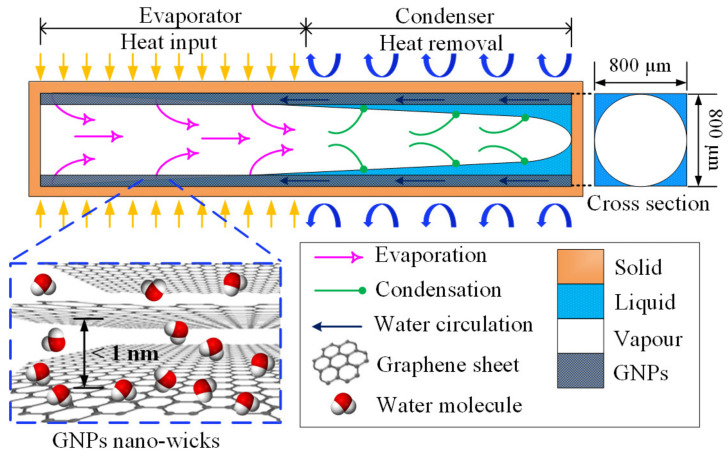
Schematic of working mechanism of a micro heat pipe with GNP nano-wicks. The inset illustrates the ultrafast transport of water molecules in the GNP nano-wicks.

This study aims to enhance the thermal performance of a water-filled micro heat pipe (MHP) by incorporating GNP nano-wicks in the microchannel of the MHP. An MHP is a promising phase-change heat-transfer device that is capable of transferring large amounts of heat energy efficiently. An MHP is an evacuated microchannel with sharp-angled corners which is partially filled with working fluid [12,13]. Unlike the macroscale wicked heat pipe, an MHP is wickless and the capillary pressure for liquid circulation is generated by the sharp-angled corners. However, the thermal performance of a heat pipe can be enhanced by incorporating composite wick structures into the heat pipe, such as porous crack wicks [49], fibrous wicks [50], grooved wicks [51,52], mesh wicks [53] and bio-wicks [54]. Here, we employ the GNPs to construct nano-wicks at the sharp-angle corners of the MHP microchannel to further enhance the capillary pressure. Generally, the operating principle of an MHP depends on two factors: the evaporation rate and the circulation rate of the working fluid. The working fluid (liquid water) absorbs the applied heat on the evaporator as the latent heat of vaporization and evaporates. The resultant water vapour then travels through the adiabatic region and arrives at the condenser section where it condenses to liquid water and the latent heat is removed from the MHP to the surroundings. The condensate is then circulated back to the evaporator section through the sharp-angled corners and the cycle is repeated. The operation of an MHP is heavily dependent on the capillary limit induced by the sharp-angled corners of microchannels. By constructing the GNP nano-wicks in the MHP, the heat-transfer rate can be enhanced significantly as the GNPs encourage film-wise evaporation and water circulation due to its ultrafast water permeation property, as depicted in Figure 1. While there are several studies highlighting the utilization of GNPs in conventional heat pipes [55,56,57,58], only a handful of studies have utilized this property of GNPs to enhance the thermal performance of MHPs by fully coating the microchannels with GNPs [7,29], achieving excellent enhancement. Most of these studies [56,57,58] applied the graphene-based nanofluids as working fluids in the heat pipes where the performance enhancement was not apparent as the enhancement is mainly dependent on the limited deposition of graphene nanoparticles on the pipe wall.

The evaporation rate and the circulation rate of water can be enhanced simultaneously by the unique characteristic of ultrafast water transport of GNPs deposited on the channel walls. In this study, for the first time, we construct the GNP nano-wicks on the inside-wall surfaces of a micro heat pipe, mimicking the micro-porous wicks in a macro-scale heat pipe. As depicted in Figure 1, the attraction of water molecules to the GNP nano-wicks is attributed to the negatively charged oxygen groups attached on the edges of graphene layers, which provide the driving force for water molecules to intercalate through the graphene layers composed of hydrophobic carbon walls. The water molecules are not in contact with the walls and they transport frictionlessly through the nanostructure. Thus, the surface-area coverage of water molecules increases significantly and the absorption of latent heat of vaporization by the water molecules escalates. Consequently, the average kinetic energy of water molecules is sufficiently high to overcome the molecular attractive forces, and this leads to remarkably enhanced evaporation rate in an MHP. At the condenser section, the circulation of condensate back to the evaporator is significantly enhanced due to the same mechanism of ultrafast water permeation of GNP nano-wicks. The effect of nano-wick thickness on the thermal performance of MHPs is investigated. This method is feasible in practical implementation to produce a highly efficient micro-scale cooling device for electronics cooling which results in better reliability and a longer lifespan of the microelectronic components for environmental sustainability.

## 2. Materials and Methods

### 2.1. Fabrication of MHP with GNP Nano-Wicks

**Figure 2:**
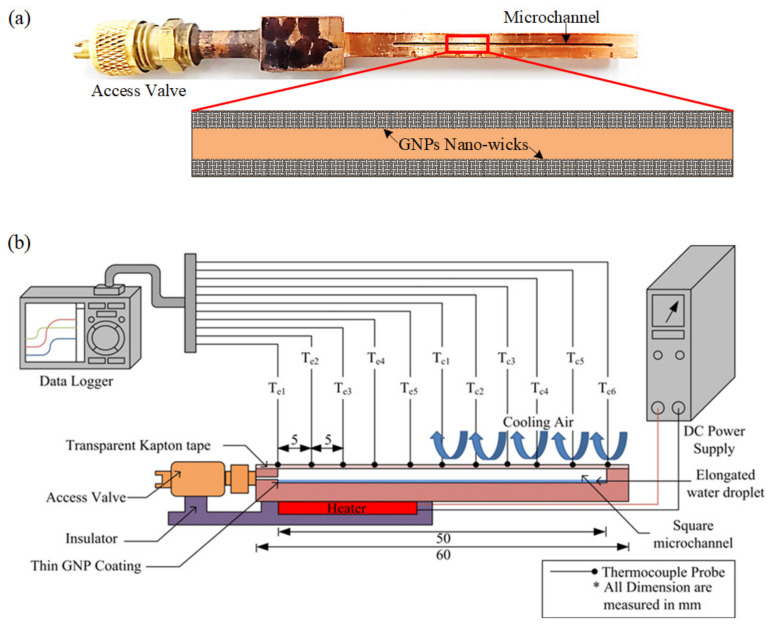
(**a**) Micro heat pipe with a microchannel grooved on a copper block. The GNP nano-wicks are constructed on the inner walls of the microchannel. (**b**) Schematic diagram of experimental setup for thermal performance evaluation of the micro heat pipe.

The conductive graphene dispersion containing GNPs of thickness less than 7 nm was purchased from Graphene Laboratories, Inc. As indicated by the materials manual, GNPs were obtained by rapidly heating the graphite intercalation compound and subsequently grinding it into fine powder form. GNPs with weight percentage of 23% were then mixed with n-butyl acetate and ethyl cellulose, followed by mild ultra-sonication treatment to ensure that the GNPs are uniformly dispersed. The MHP, as depicted in Figure 2a, is made of copper due to its high thermal conductivity, (*k* = 388 W/m∙K). A square microchannel of 800 µm × 800 µm with a length of 50 mm is micro-machined into copper using computer numerical control (CNC). The microchannel is sealed at one end while the other end is connected to an access valve for air evacuation and charging of the working fluid. To form the nano-wicks on the inner walls of the microchannel, as illustrated in Figure 2a, a total of 5 µL of dispersed GNPs deposited onto the inner wall of the MHP with a high-precision micropipette, while a level rule is used to ensure the MHP is positioned horizontally such that uniform GNP deposition is achieved. Subsequently, the GNP-coated MHP undergoes a thermal curing process at 250 °C for 30 min to completely functionalize the GNPs. After that, a thin layer of GNP nano-wicks is formed on the inner walls. Nitrogen gas is then utilized for surface cleaning. The thickness of the GNP wick is varied by repeatedly coating the surface with the above method. De-ionized (DI) water is chosen as working fluid due to its superior properties such as high surface tension, high latent heat of vaporization and low viscosity. In this study, three specimens with different wick thicknesses of 27.8 µm, 34.7 µm and 49.6 µm, are fabricated. An amount of 6.4 µL of DI water, equivalent to 20% of the working fluid charge ratio, is charged into the microchannel. The top part of the microchannel is then sealed with a thin layer of adhesive polyimide film. To prevent exudation of working fluid from the microchannel during evacuation, the liquid water inside the microchannel is frozen in a freezer at a temperature of −40 °C for 30 min. After the working fluid has solidified, the specimen is evacuated through a one-way valve under a pressure of 0.2 Pa. Once the desired vacuum condition is achieved, the MHP is detached from the vacuum system to cut off the vacuum passage. The specimen is then ready to be tested after the frozen water liquefies at the room temperature.

### 2.2. Experimental Setup

The schematic diagram of the experimental setup is depicted in Figure 2b. The MHP is oriented in a horizontal position such that the gravity effect can be neglected. A flat heater is attached to the base of the specimen (20 mm in length) which is considered as the evaporator section while the remaining portion (30 mm) is considered as the condenser section. Thermal grease is applied between them to reduce the thermal contact resistance. In order to minimize heat loss, the heater is encased in wood fiber and covered with a layer of Teflon insulation blanket. The radiation heat transfer is neglected as the surface temperature is relatively low. The plate heater is powered and regulated using a standard DC power supply unit (PS 8160-04T, EA Elektro-Automatik, Viersen, Germany) with a measurement uncertainty of ±0.5%. Eleven T-type thermocouples are attached onto the copper surface in order to measure the surface temperature distribution at every increment of 5 mm, as shown in Figure 2b. A thermocouple is also placed near the vicinity of the MHP to measure the ambient temperature, which is maintained at 24 °C with fluctuation of ±1.5 °C. The temperature is recorded by a MIDI data logger (GL820, Graphtec Co., Yokohama, Japan) over a period of 45 min to ensure that the MHP has reached its steady state. To ensure the repeatability of the experimental results, each experiment run is repeated at least 3 times and the error bars are plotted on the data points with 95% confidence level. At 95% confidence level, the error can be estimated as

(1) $$\mathrm{error}=1.96\left(\frac{\sigma }{\sqrt{n}}\right)$$

where σ is the standard division and *n* is the number of experiment runs.

### 2.3. Evaluation of Water Permeation in GNP-Coatings

In order to observe and quantify the water permeability of GNPs, the changes in contact angle of water droplets on copper and GNP surfaces are measured by a high precision goniometer (Ramé-hart 250-F1 Tensiometer with DROPimage Advance). The experiments are conducted at a room temperature of 25 °C and a standard atmospheric pressure. Measurement of its static contact angle would provide the water permeability of GNPs over time. DI-water droplet (3 µL) is dropped onto the tested surface and the variations in static contact angle for each surface are recorded over a period of 25 s. In addition, a small amount of fluorescent dye is added into DI-water and the trace of the fluorescent dye is recorded by a Nikon Eclipse CI-E microscope with Epi fluorescent attachment (Nikon Instrument Inc, New York, NY, USA). With the use of fluorescent dye, the spreading and permeation of the water droplet into the GNP surface can be identified visually and qualitatively. The digital images are then processed using the Mathematica software [59] where the intensity fluxes of the fluorescent domains on the surfaces can be quantified. The experiment is repeated 5 times for each surface to ensure experimental consistency.

### 2.4. Characterization of GNP Coatings

Multiple 5 mm cut sections of MHP channels are made through delicate CNC machining. The surface morphology of GNPs and the thickness of the nano-wick are characterized and measured using SU-8010 FESEM (Hitachi Ltd., Tokyo, Japan). The X-Ray diffraction (XRD) data is obtained using an X-ray diffractometer (D8 Discover, Bruker, Billerica, MA, USA) with Cu Kα (λ = 1.54056 Å) and a scanning rate of 0.02° s^−1^ for the range between 5° to 50°. The samples are also characterized by X-ray photoelectron spectroscopy (XPS) using PHI Quantera II (ULVAC-PHI. Ltd., Chigasaki, Japan) with a monochromatic Al Kα (hv = 1486.6 eV) X-ray source (with a beam diameter of 100 μm) operated at 25.1 W. The XPS survey (wide) scan utilizes a pass energy of 280 eV with 1 eV per step and the narrow scan analysis consumes a pass energy of 112 eV with 0.1 eV per step for the chemical state analysis. The Raman spectroscopy is conducted using Horiba LabRAM HR-Evolution (Horiba Scientific, Kyoto, Japan) with a 514-nm laser.

### 2.5. Thermal Performance Evaluation of MHP

The thermal performance of a capillary-driven two-phase device is governed by the circulation effectiveness and the phase-change heat transfer of the working fluid. Here, we utilize two different indicators to quantify the thermal efficiency, i.e., the effective thermal conductance, *k*_eff_, and the effectiveness of the MHP on the heated surface, *U*_h_. A higher thermal conductance indicates a higher overall performance and a larger value of the effectiveness of MHP indicates higher heat dissipation from the heated surface. The effective thermal conductance is obtained by evaluating the axial temperature drop from evaporator to condenser, $\Delta T=T_{e}-T_{c}$ for a particular heat load, where *T*_e_ is the average temperature of the evaporator section and *T*_c_ is the average temperature of the condenser section. The effective thermal conductance is evaluated as

(2) $$k_{\mathrm{eff}}=\frac{{\dot{Q}}_{\mathrm{in}}}{\Delta T}\frac{L_{\mathrm{eff}}}{A_{c}}$$

In Equation (2), ${\dot{Q}}_{\mathrm{in}}$ is the applied heat load which is calculated based on the principle of energy conservation, whereby the heat dissipated from the condenser section is equal to the net heat transfer across the cooling device. The effective length is given by $L_{\mathrm{eff}}=0.5\left(L_{e}+L_{c}\right)$ [7,29] and *A*_c_ is the cross-sectional area of the microchannel.

The effectiveness of the MHP is the ratio between the ratio of the cooling effect of a charged MHP and the case without MHP (benchmark) and is used to quantify the heat dissipation from the heated surface for different configurations of the MHP. The overall heat transfer coefficient of a charged MHP on the heated surface is given by $U_{h}={\dot{Q}}_{\mathrm{in}}/A_{h}\left(T_{h}-T_{\infty }\right)$, where $T_{\infty }$ is the ambient temperature and *A*_h_ is the heated surface area. During the experiments, the heat load is kept constant and the effectiveness of the MHP can be expressed as [7]

(3) $$\eta =\frac{U_{h}}{U_{h,o}}=\frac{T_{h,o}-T_{\infty }}{T_{h}-T_{\infty }}$$

where $U_{h,o}$ and $T_{h,o}$ are the heat transfer coefficient and the heated surface temperature for the case without MHP, respectively. To investigate the effectiveness of the MHP on the heated surface, *η* is the relative comparison of the heat transfer coefficients by using the value of the no-cooling-device case as the basis for comparison. The heat transfer is enhanced when *η* is larger than unity and vice versa.

## 3. Results and Discussion

### 3.1. Wetting Behavior of GNP Coatings

**Figure 3:**
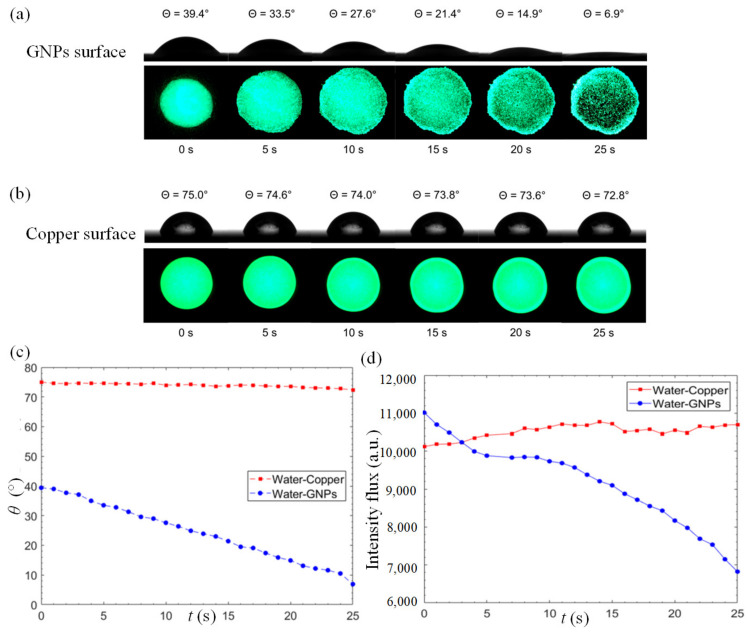
Time-lapse goniometry images of water droplet contact angle and digital images of fluorescence-dyed water droplet on the (**a**) GNPs, and (**b**) copper surfaces. (**c**) Static contact angle of a 3 μL fluorescent water droplet on the GNPs-coated and the copper surfaces as a function of time. (**d**) Intensity flux of a 3 μL fluorescent water droplet on the GNP-coated and copper surfaces as a function of time.

To gain a better insight on the excellent water permeation of GNP coatings, we compared the variation of static contact angle on the GNP-coated surface and a reference copper surface. Figure 3a,b illustrate the reduction in static contact angle and the changes in fluorescence-dye intensity flux on the surface over a period of 25 s for the GNPs-coated and copper surfaces, respectively. As depicted in Figure 3a, the anomalous rapid water permeation of GNPs leads to a rapid decrement in static contact angle from 39.4° to 6.9° within a short timeframe, leading to a reduction rate of 1.3°/s. Apart from the high reduction rate, the initial static contact-angle measurement of a GNP–water interface manifests its high surface-wettability property. At the same time, the fluorescent analysis reveals a clear increment in the wetted area between 0 s ≤ t ≤ 10 s. For t > 10 s, a significant decrement in fluorescent intensity is observed as more water molecules intercalate into the GNP nanostructures. An interesting observation at the edge of the fluorescent domain reveals that water tends to flow outwards into the GNP layer in an omnidirectional manner. In Figure 3b, a minor change in static contact angle over time is observed on the copper surface with a relatively high initial contact angle at 75° which only marginally decreases to 72.8° over a period of 25 s, with a reduction rate of 0.088°/s. The reduction rate at the GNP surface is 14.7 times of that of the copper surface. The fluorescence intensity of the copper surface remains invariant with insignificant changes over time.

**Figure 4:**
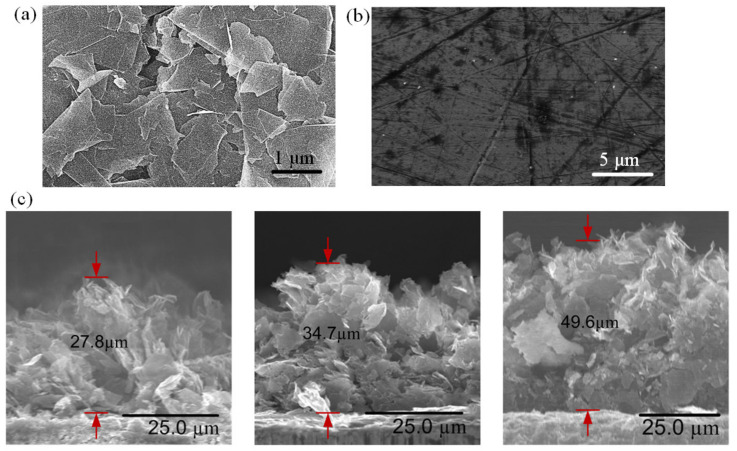
FESEM images of (**a**) GNP-coated, and (**b**) uncoated copper surfaces. (**c**) FESEM images of cross-sectional view of GNP nano-wicks with thicknesses of 27.8 µm, 34.7 µm, and 49.6 µm.

Figure 3c depicts the static contact angle of a 3 μL fluorescent water droplet on the GNP-coated and copper surfaces as a function of time. In the case of GNP-coated surface, a discernible and steady decrement in static contact angle can be observed while the changes on the copper surface are negligible. The fluorescence intensity flux is used as a quantitative indicator of water permeation, as shown in Figure 3d. The intensity flux of the copper surface manifests insignificant differences over time. On the other hand, a clear decrement of intensity flux is observed on the GNP surface, indicating that the water molecules intercalate into the graphene layers. From Figure 3d, three stages of water permeation can be observed. From 0 to 5 s, the fluorescent domain starts to spread into a larger area due to the hydrophilic nature of the GNP surface. Between 6 s and 12 s, the expansion in area slows down as water molecules start to permeate into the micro-porous structure formed by the stacked nanoplatelets, as depicted in Figure 4a. From 12 s onwards, the water molecules intercalate frictionlessly into nanochannels formed by the graphene layers (each individual graphene nanoplatelet consists of a few graphene layers functionalized with oxygenated groups), as depicted in the inset of Figure 1. This leads to the ultrafast water permeation phenomenon where the fluorescence intensity flux decreases rapidly as shown in Figure 3d. The oxygenated functional groups such as hydroxyl, epoxy, carbonyl and carboxyl groups play their critical role in propelling the water molecules through the graphene nanochannels [44,45,46,47]. The smooth hydrophobic carbon-walled layers allow near-frictionless flow of water molecules, and the oxygenated functional groups engender driving force for fast water permeation across the hydrophobic carbon walls. As a result, a series of hydrophilic–hydrophobic patterned surfaces are formed and promote the ultrafast water permeability of GNP surfaces.

### 3.2. Characterization Analysis of GNPs

The surface morphology of GNPs was characterized using field emission scanning electron microscopy (FESEM). The morphological image of the GNPs in Figure 4a reveals a coarse surface with graphene nanoplatelets randomly stacked on top of each other while the edges of the nanoplatelets are distinctive. The average diameter of the graphene nanoplatelet is 1–2 μm, and the average thickness is approximately 7 nm. As the solvent is removed during the thermal curing process, the GNP interactions are governed by the Van de Waals forces among the platelets, resulting in a graphite-like structure with an abundant distribution of micro-sized cavities which provide passages for water molecules to flow among the GNPs. The water permeation and intercalation will be discussed further in the later section. The FESEM image of an uncoated copper surface is shown in Figure 4b as a comparison. The copper surface manifests a smooth and non-porous surface. The nano-wicks formed at the inner-wall surfaces of the microchannels manifest in three different thicknesses. The average thickness δ can be approximated from the FESEM cross-section images, as depicted in Figure 4c. In this study, nano-wicks with three different thicknesses, i.e., 27.8 µm, 34.7 µm, and 49.6 µm, are constructed.

**Figure 5:**
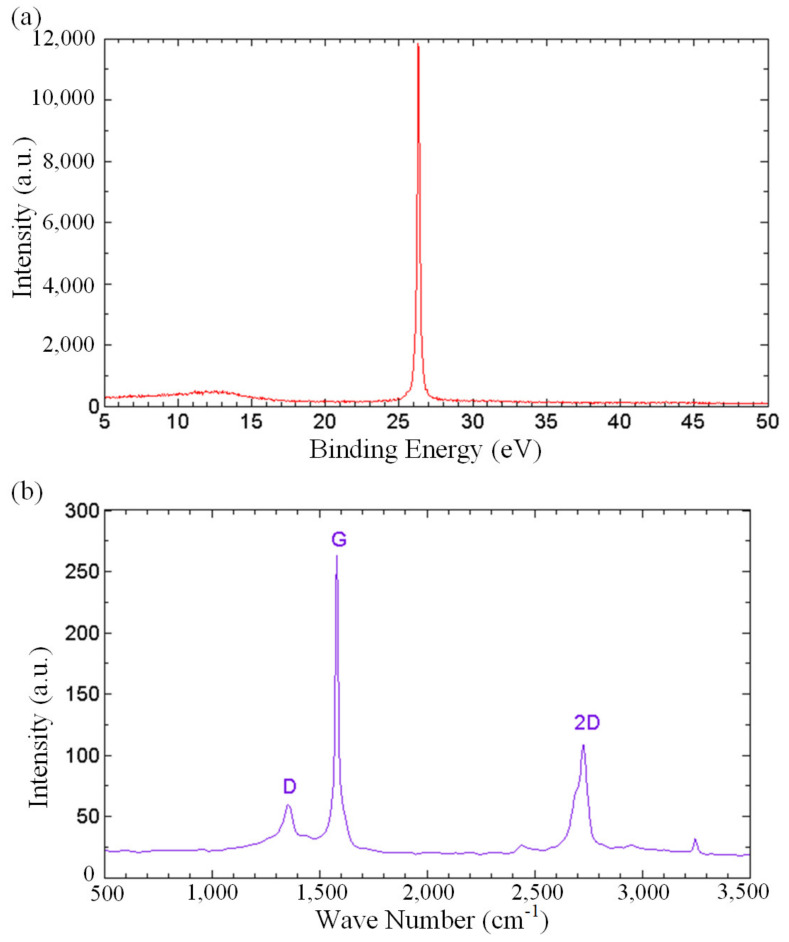
(**a**) XRD spectrum, and (**b**) Raman spectrum of the thermally cured GNPs.

In order to gain more understanding on the role of oxygenated functional groups in fast water permeation of GNPs, spectroscopic analyses were carried out. Figure 5a depicts the X-ray diffraction (XRD) spectrum of the thermally cured GNPs. The diffraction peaks at 2θ angles of 26.3° in the XRD pattern corresponds to the well-ordered graphitic structures. Bragg’s law was used to calculate the interlayer distance, showing a value of 3.39 Ǻ. The XRD spectrum substantiates that the carbon bonds originated from an unambiguous crystal network of graphene, attesting to the unaltered crystalline structure of carbon under the functionalization during the thermal curing process. Raman spectroscopy was performed on the GNP-coated surface to analyze the degree of defects in the GNPs, as shown in Figure 5b. The Raman spectrum shows the D band at 1354 cm^−1^, G band at 1581 cm^−1^ and the 2D band at 2726 cm^−1^. D band is a representation of structural defects associated with vacancies, grain boundaries and amorphous carbon species, while G bands relate to the sp^2^ graphitic domains [60,61]. The small value of the intensity ratio of D and G bands, I_D_/I_G_ = 0.23, indicates a minimal structural defect, revealing that the non-oxidized regions are dominant in the GNPs.

**Figure 6:**
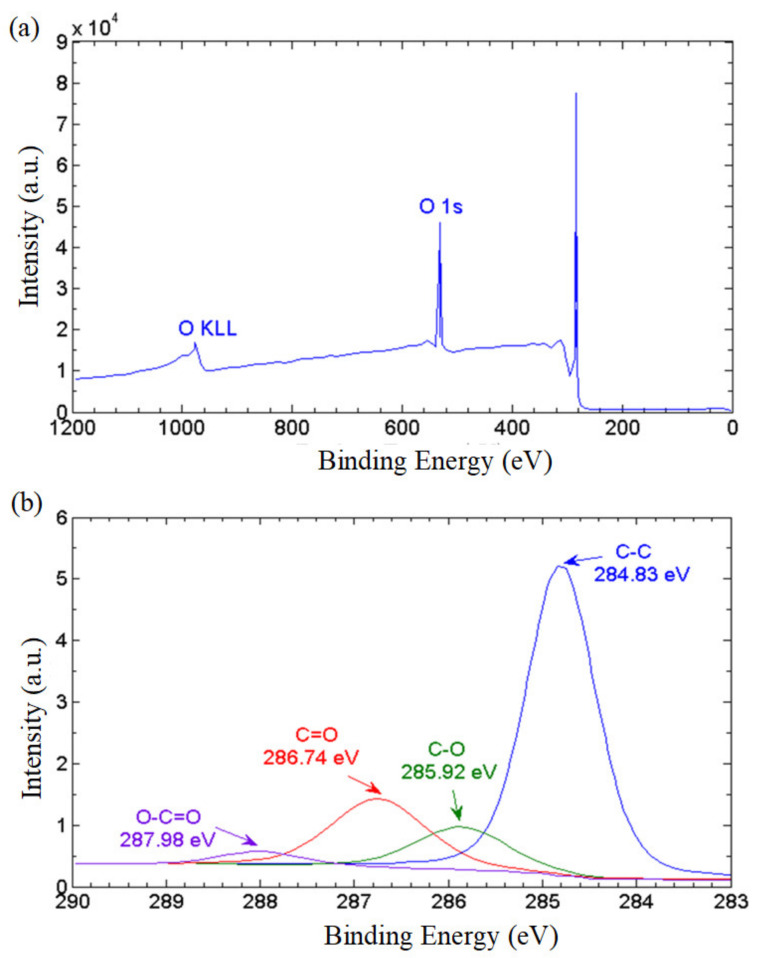
(**a**) XPS survey spectrum, and (**b**) XPS carbon (C1) spectra of the thermally cured GNPs.

To further elucidate the ratio of the non-oxidized regions to the oxidized regions, we conducted X-ray photoelectron spectroscopy (XPS). The XPS wide-scan (survey) spectrum in Figure 6a reveals that the GNPs consist of 87.45% carbon, and 12.55% oxygen based on atomic concentration, with a carbon to oxygen ratio of 6.96. The XPS narrow scan conducted on carbon bonds (C1s) revealed the population of oxygenated functional groups attached on the graphene nanostructures. As depicted in Figure 6b, the primary spectrum manifests a peak of graphitic C–C bond at 284.83 eV. The functional epoxy (C–O–C), carboxyl (O=C–OH) and hydroxyl (C–OH) groups are identified in the spectra by the peaks of 289.98 eV, 286.74 eV and 285.92 eV, respectively. The population of the hydrophilic functionalities can be estimated by evaluating the area under the respective curves. The non-oxidized region represented by the C–C bond accounted for a majority of 64.96% while the epoxy, carboxyl and hydroxyl groups constituted 19.64%, 3.41% and 11.98%, respectively, of the total bonds. This implies that the GNP nanostructure has substantial non-oxidized regions providing frictionless nanocapillaries for the effective transport of water molecules. On the other hand, the oxygenated functional groups provide a continuous attraction force that propels the water molecules into the nanochannels formed between the graphene layers, and a series of hydrophobic and hydrophilic networks are formed to achieve the water-driving mechanism. This leads to a remarkable superhydrophilicity with high water permeability on the GNP surface.

**Figure 7:**
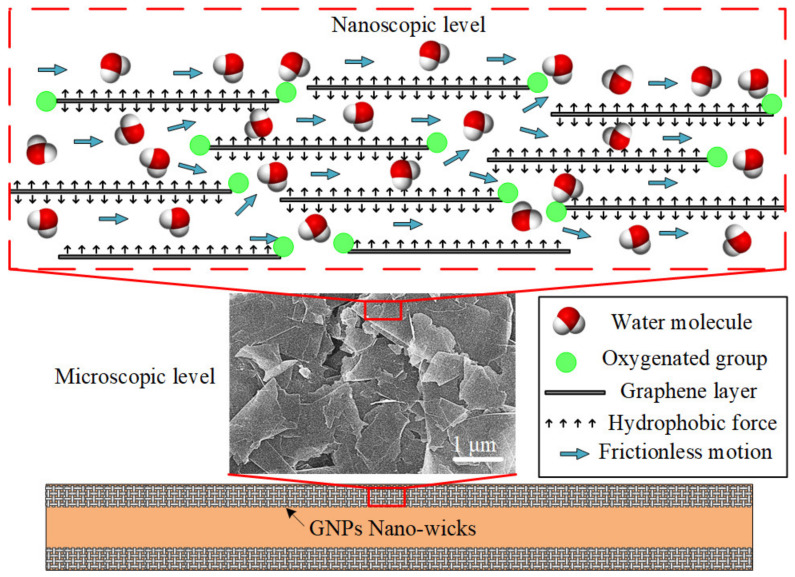
Schematic depicting the nano-wicks formed on the inner-wall surface of a microchannel. At the microscopic level, the platelet-by-platelet structure of GNPs is observed. At the nanoscopic level, each nanoplatelet consists of several graphene layers of which the gaps between them form a network of nanocapillaries.

The ultrafast water permeation property of GNPs is well-suited to be applied in a phase-change heat-transfer device, especially in the micro heat pipe (MHP). In this study, the inner-wall surfaces of the microchannel are coated with a thin layer of GNPs to imitate the nano-wicks of the heat pipe, as depicted in Figure 7. At the microscopic level, the platelet-by-platelet structure of GNPs is observed. The average thickness of an individual GNP is 7 nm, with a lateral sheet diameter of approximately 1–2 μm. The randomly stacked graphene nanoplatelets form plentiful micro-sized cavities which supply ample space for water permeation. At the nanoscopic level, each nanoplatelet consists of several graphene layers of which the gaps between them form a network of nanocapillaries where the layers of hexagonal arrangements of carbon atoms are denoted as graphene layers. The negatively charged oxygenated functional groups, such as hydroxyl, carbonyl, and carboxyl, are attached at the edges of the graphene layers. The polar water molecules are attracted and propelled through the frictionless hydrophobic nanochannels formed by the graphene layers. This expedites the transport of water molecules from the condenser section to the evaporator section due to the nanocapillary pressure induced by the GNP nano-wicks. It has been theoretically proven that the nanocapillary pressure is at least four orders of magnitude greater than that of the microscale capillary pressure [7,62]. The water molecules adjacent to the graphene sheets experience near frictionless flow as the nanocapillary pressure drives them through the hydrophobic graphene sheets. The ultrafast water permeation of GNPs is attributed to the frictionless interaction between the water molecules and the carbon wall as well as the hydrophilic attraction force of oxygenated functional groups. Molecular dynamics simulations have been conducted to elucidate the ultrafast water permeation mechanism of GNPs [44,46,48]. Through the nanocapillary pressure induced by the GNP nano-wicks, the water molecules are expediated from the condenser section to the evaporator section. As the water molecules intercalate and spread through the graphene nanostructure, they transform in shape to a thin film and the surface area covered by the water molecules significantly increases, enabling the absorption of the latent heat of vaporization at the evaporator section. Subsequently, the average kinetic energy of the water molecules increases beyond the attractive intermolecular forces and the liquid water transforms into vapor water, inducing a remarkably enhanced evaporation rate [63].

## 4. Discussion

**Figure 8:**
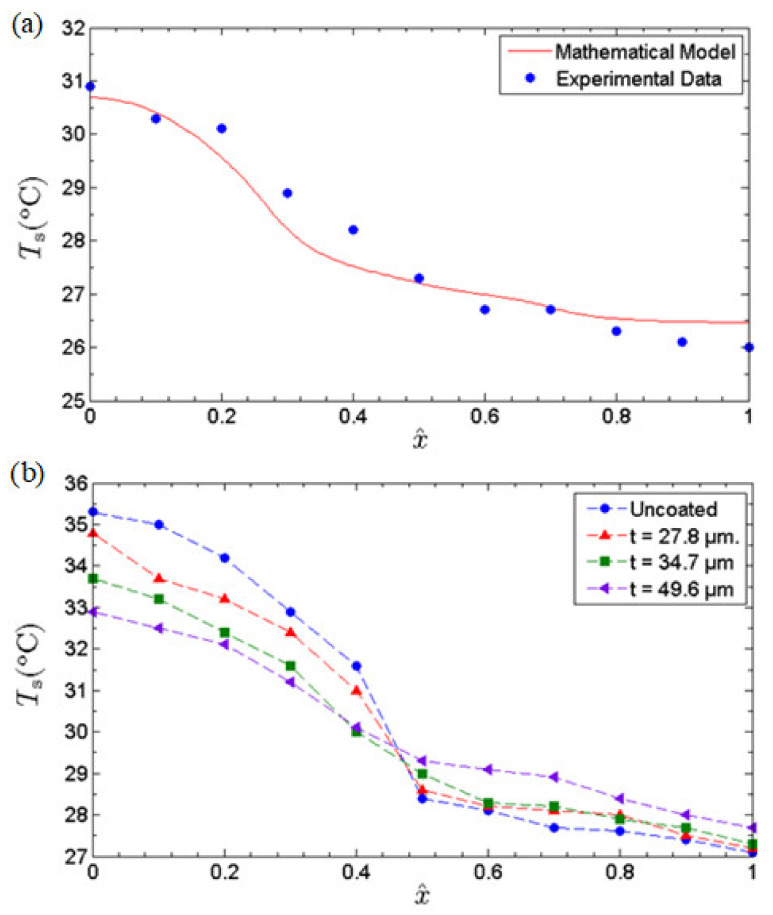
(**a**) Comparison of axial solid wall temperature profile of the current experimental investigation with 20% charge ratio (uncoated case) against the theoretical prediction based on a mathematical model developed by Gan et al. [12] at heat input of 1.1 W. (**b**) The axial temperature profiles of the uncoated MHP and MHPs with different thicknesses of nano-wicks at a heat input of 1.6 W.

In view of the anomalous fast water permeation characteristic of GNPs, we investigated the thermal performance enhancement of an MHP whose inner-wall surfaces were coated with GNP nano-wicks. The effects of the thickness of GNP nano-wicks on the thermal performance of MHP were elucidated. Three different thicknesses of GNP nano-wicks, i.e., 27.8 μm, 34.7 μm, and 49.6 μm, were studied. To assure the accuracy of the experiments, we benchmarked the experimental data of the uncoated case with the mathematical model developed in our previous study [12]. In the mathematical model, water was used as the working fluid and identical geometrical parameters were applied. Under a heat input of 1.1 W, the axial temperature distribution was plotted against the experimental data obtained. As depicted in Figure 8, the experimental result agrees well with the mathematical model, both quantitatively and qualitatively.

To gain an insight into the effects of GNP nano-wicks, the axial temperature profiles at a heat input of 1.6 W were obtained and depicted in Figure 8a for all 3 MHPs with different thicknesses of nano-wicks along with the uncoated MHP as a benchmark. The axial temperature profiles reveal that the incorporation of GNP nano-wicks makes the axial temperature profiles more uniform. It can be observed that the MHP with a GNP nano-wick thickness of 49.6 μm has a temperature difference, ΔT, of 3.2 °C compared to that of the uncoated MHP of 6.1 °C, where temperature difference, ΔT, is defined as the difference between the average evaporator temperature and the average condenser temperature. The decrease in the temperature difference is essentially attributed to the enhancement in both evaporation strength and the water circulation rate of the MHP. The enhancement in evaporation strength is evidently shown in the evaporator section of the MHP, where the evaporator temperatures decrease with increasing thickness of the nano-wicks. This is attributed to the ultrafast water permeation of GNP nano-wicks, leading to the formation of ultrathin water liquid film at the evaporator section and encouraging film-wise evaporation. With the increase in heat-transfer surface area, the ultrathin liquid film evaporates almost instantly as it approaches the heated surface. In addition, a slight increase in condenser temperature profile is observed, attributed to the increase in heat-transfer surface area provided by the nano-wicks, which consequently improves the rate of condensation. The water condensate travels back to the evaporator section through the nanocapillary networks of GNPs.

**Figure 9:**
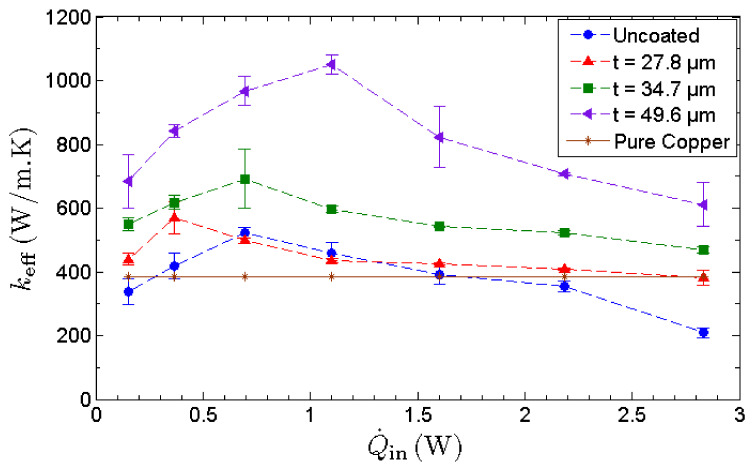
Effective thermal conductance, *k*_eff_, of MHPs as a function of heat input. The thermal conductivity of copper is used as a benchmark.

The effective thermal conductance, *k*_eff_, is used as a performance indicator to quantify the thermal performance of an MHP. As depicted in Figure 9, the effective thermal conductance of all MHPs manifests an inverted-V shaped trend with the increasing heat input. Initially, the effective thermal conductance increases with heat input as the water circulation rate is more than capable of overcoming the rate of evaporation. Hence, the MHP is underloaded and operates within its operating limit. When the heat input is increased, the evaporation rate intensifies and eventually dryout occurs as the water circulation rate fails to supply sufficient water for the intense evaporation at the evaporator section. The lack of working fluid in the evaporator section retards the phase-change heat transfer, inducing a high temperature at the evaporator and causing a drastic drop in the effective thermal conductance. At the optimum condition, the effective thermal conductance of the MHP with the thickest nano-wicks achieves a maximum value of 1050 W/m⋅K, manifesting an enhancement of 128% compared to that of the uncoated MHP.

It has been pointed out that the improvement in heat transfer is attributed to the enhanced thermal conductivity of graphene, based on the miraculously high thermal conductivity of a single layer of pristine graphene [1,5]. However, GNPs are commonly synthesized through scalable methods such as thermal and chemical reduction of graphene oxide or thermal exfoliation of expandable graphite. These methods yield significantly lower thermal conductivity compared to that of pristine graphene. As the layered structure of graphene is governed by weak cross-plane van der Waals bonding, the cross-plane heat transfer is ineffective, causing a drastic drop in thermal conductivity [64]. Therefore, in this study, the increase in effective thermal conductance of MHP with nano-wicks is not induced by the exceptionally high thermal conductivity of graphene but by the anomalous fast water permeation property of the GNP nanostructure. This is evident as the thermal conductance increases with the thickness of GNP nano-wicks. The heat input corresponding to the maximum thermal conductance is denoted as the heat transport capacity of MHP. Furthermore, it can be observed that the heat transport capacity of MHPs with nano-wicks increases with increasing thickness of GNP nano-wicks. As the thermal performance of MHP is essentially governed by evaporation, condensation and water circulation, the ultrafast water permeation property of GNPs benefits these processes in a significant manner. At any instant, the thin water film formed within the graphene nanostructures continuously draws liquid water from the condenser to the evaporator to sustain the high evaporation rate. Despite the decrease in the thermal conductivity of multilayer graphene, a thicker GNP nano-wick leads to an increase in the number of nanocapillaries for the intercalation of water molecules through the nanocapillary network. This in turn improves the water circulation rate and results in a much higher capillary limit of MHP.

**Figure 10:**
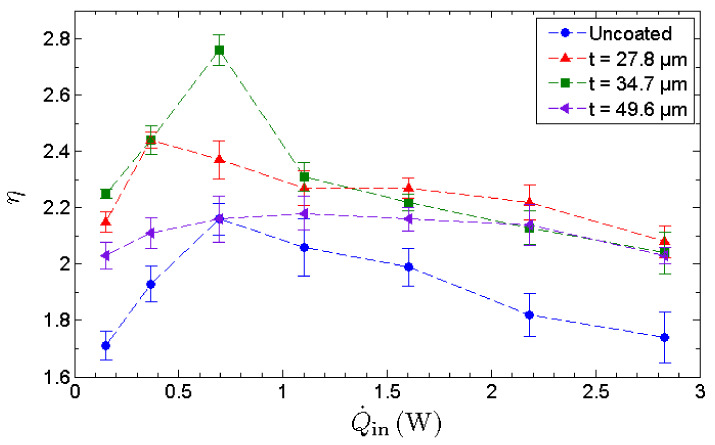
Heat-transfer enhancement ratio of an uncoated MHP along with 3 separate GNP-coated MHPs with coating thicknesses of 27.8 μm, 34.7 μm and 49.6 μm, respectively.

To assess the evaporation strength of the evaporator section, the effectiveness of MHP, η, is utilized. The effectiveness of the MHP is a performance indicator evaluated based solely on the cooling capability of the MHP on the heated surface as shown in Figure 10. The effectiveness exhibits an inverted V-trend that indicates the existence of an optimal operating condition. The effectiveness increases with the heat input up to its maximum allowable heat input, whereby dryout occurs and decreases beyond this point. It is observed that the optimal thickness of a GNP nano-wick is 34.7 µm rather than 49.6 µm, indicating that the evaporation strength is not proportional to the thickness of nano-wick. As benchmarked with the uncoated MHP, the MHP with a 34.7 µm nano-wick manifests a maximum enhancement of 32% in terms of its cooling capability on the heated surface. The enhancement of evaporation is due to the formation of ultrathin film of water where the film-wise evaporation is induced. In this case, a thinner GNP nano-wick is more favorable for the film-wise evaporation. However, a thicker GNP nano-wick promotes a higher water circulation rate from the condenser to the evaporator. Thus, to compensate for the film-wise evaporation that favors an ultrathin nano-wick, there exists an optimal thickness of GNP nano-wick, i.e., 34.7 µm, which leads to the maximum evaporation rate. The effective thermal conductance indicates the overall thermal performance of MHP where the evaporation rate and the water circulation rate are taken into account. On the other hand, the effectiveness of the MHP solely measures the evaporation strength which is enhanced significantly due to the formation of an ultrathin film of water in the GNP nanostructures. In conclusion, the GNP nano-wicks enhance both the evaporation and the condensate circulation processes of an MHP, and hence significantly enhance the thermal performance of an MHP as well as the cooling effect on the heated surfaces.

## 5. Conclusions

In this study, we constructed GNP nano-wicks on the inside-wall surfaces of a micro heat pipe, mimicking the micro-porous wicks in a macro-scale heat pipe. Based on the performance indicators, we conclude that the use of GNP nano-wicks manifests a significant improvement in the phase-change heat transfer attributed to the unique ultrafast water permeation property of GNPs based on the following mechanisms:

(1) The hydrophobic, atomically smooth carbon walls of GNPs nanostructures provide a network of nanocapillaries that allows water molecules to intercalate among the graphene layers and travel in a near-frictionless manner.
(2) Together with the attraction force of the oxygenated functional groups, a series of hydrophobic and hydrophilic surfaces are formed that significantly improve the water circulation rate.
(3) At the same time, the intercalation of water molecules encourages the formation of a water-thin film that leads to a larger heat-transfer surface area. In turn, film-wise evaporation occurs and much higher latent heat is absorbed and transferred to the condenser section.
(4) Simultaneously, the nanocapillary pressure along with the oxygenated functional group drives the condensate back to the evaporator section in a continuous and efficient manner. The GNP nano-wicks provide significant enhancement in the key processes of an MHP, namely the evaporation and the condensate circulation, leading to a remarkable improvement in its heat transfer efficiency which is evidently dependent on the thickness of the GNP nano-wicks.

The effective thermal conductance of the MHP with the thickest nano-wicks (49.6 μm) achieves a maximum value of 1050 W/m·K, manifesting an enhancement of 128% compared to that of the uncoated MHP. On the other hand, it is observed that the optimal GNP nano-wick thickness for the effectiveness of the MHP is 34.7 µm rather than 49.6 µm, indicating that the evaporation strength is not proportional to the thickness of the nano-wick. The enhancement of evaporation is due to the formation of an ultrathin film of water where the film-wise evaporation is induced. In this case, a thinner GNP nano-wick is more favorable to the film-wise evaporation. However, a thicker GNP nano-wick promotes a higher water circulation rate from the condenser to the evaporator. To compensate for the film-wise evaporation that favors an ultrathin nano-wick, there therefore exists an optimal thickness of GNP nano-wick, i.e., 34.7 µm, which leads to the maximum evaporation rate. This study provides important insights into the feasibility of incorporating GNP nano-wicks into the MHP and elucidated the effects of the thickness of GNP nano-wicks on the thermal performance of MHP. GNP nano-wicks enhance both the evaporation and the condensate circulation processes of an MHP and hence significantly enhance the thermal performance of an MHP as well as the cooling effect on the heated surfaces.

## Data Availability

The data presented in this study are available on request from the corresponding author. The data are not publicly available as the data also form part of an ongoing study.
